# Bladder Cancer highlighted

**DOI:** 10.1590/S1677-5538.IBJU.2020.02.01

**Published:** 2020-01-10

**Authors:** Luciano A. Favorito

**Affiliations:** 1 Brasil Editor in Chief, Int Braz J Urol,Brasil; 2 Universidade do Estado de Rio de Janeiro Rio de JaneiroRJ Brasil Professor Associado da Unidade de Pesquisa Urogenital - Universidade do Estado de Rio de Janeiro - Uerj, Rio de Janeiro, RJ, Brasil; 3 Hospital da Lagoa Federal Rio de JaneiroRJ Brasil Serviço de Urologia, Hospital da Lagoa Federal, Rio de Janeiro, RJ, Brasil

The March-April number of *Int Braz J Urol*, the second under my supervision, presents original contributions with a lot of interesting papers in different fields: Prostate Cancer, Renal Cell Carcinoma, Penile trauma, Bladder Cancer, BPH, Laparoscopy and Testicular Cancer. The papers came from many different countries such as Brazil, USA, Turkey, China, Korea, Colombia, India and Italy, and as usual the editor ´s comment highlights some of them. In the present issue we present two important reviews: in page 152 Dr. Barros and colleagues from Brazil (1) review in a nice narrative the urethral injury in penile cancer, and Dr. Li and colleagues from China (2) present in page 158 an important meta-analysis about preoperative serum total cholesterol is a predictor of prognosis in patients with renal cell carcinoma, but the bladder cancer is the highlight of this number.

Dr. Hamad and collegues (3) supervised by Dra. Angela Smith of University of North Carolina – USA performed on page 169 a elegant review about bladder preservation therapies, patient selection criteria, and functional and oncologic outcomes for Bladder preservation therapies in muscle-invasive bladder cancer and concluded that he breadth of strategies that aim to preserve a patient’s bladder while still optimizing local tumor control and overall survival and future areas for innovation include the use of predictive biomarkers and implementation of immunotherapy. The editor would like to highlight the following works too:

Dr. Ölçücü and collegues from Turkey (4) on page 185 evaluate the effects of solifenacin, darifenacin, and propiverine on nasal-, subfoveal-, temporal choroidal thicknesses (NCT, SFCT, TCT), intraocular pressure (IOP) and pupil diameter (PD) in a 165 patients with overactive bladder (OAB) and concluded that solifenacin significantly reduced IOP, darifenacin significantly reduced NCT and propiverine significantly increased PD in patients with OAB who had normal ophthalmologic examinations after the twelve weeks of treatment. These findings can help to decide appropriate anticholinergic drug choice in OAB medical treatment for patients with eye-related disorders.

Dr Arda and Collegues (5) from Turkey perfomed on page 216 in a interesting determined the utility of preoperative complete blood count (CBC) based systemic inflammatory markers in the prediction of testicular cancer and its prognosis in 182 patients and concluded that several systemic inflammatory markers, which are obtained by routinely performed cost-effective blood tests, could demonstrate incremental predictive and prognostic information adjuvant to preoperatively achieved testicular tumor markers.

Dr. Lal and Collegues (6) from India performed on page 234 the study that is on the cover in this number. The authors assessed the role of MRI for predicting postoperative renal function by preoperative estimation of renal parenchymal volume and correlation with glomerular filtration rate (GFR) in 30 patients with median tumor volume of 175.7cc. They concluded that preoperative residual parenchymal volume on MRI renal volumetry correlates well with postoperative GFR in patients with RCC undergoing radical nephrectomy or nephron sparing surgery.

Dr. Tae and Collegues (7) from Korea studied on page 244 the usefulness of natural killer cell activity (NKA) in diagnosing of prostate cancer (PC) in 102 patients and concluded that low NKA and high PSA levels were likely to be associated with a positive TRBx outcome. NKA detection was easy and improved the diagnostic accuracy of prostate cancer.

Dr. Reis Leonardo and Collegues (8) from Brazil shows on page 253 the role of laparoscopic pyeloplasty (LP) in children is less well defined and has slowly emerged as an alternative procedure in 38 children and suggested that LP has good functional and cosmetic results, not compromising the success of the open procedure, regardless patient’s age.

We hope that readers will enjoy the present number of the *International Brazilian Journal of Urolog*y.
